# Perturbation of Nuclear Hormone Receptors by Endocrine Disrupting Chemicals: Mechanisms and Pathological Consequences of Exposure

**DOI:** 10.3390/cells9010013

**Published:** 2019-12-19

**Authors:** Julie M. Hall, Callie W. Greco

**Affiliations:** Frank H. Netter MD School of Medicine, Department of Medical Sciences, Quinnipiac University, NH-MED, North Haven, CT 06473, USA; callie.greco@qu.edu

**Keywords:** nuclear hormone receptors, endocrine disrupting chemicals (EDCs), estrogen receptor, estrogens, exposure, receptor activation, cancer

## Abstract

Much of the early work on Nuclear Hormone Receptors (NHRs) focused on their essential roles as mediators of sex steroid hormone signaling in reproductive development and function, and thyroid hormone-dependent formation of the central nervous system. However, as NHRs display tissue-specific distributions and activities, it is not surprising that they are involved and vital in numerous aspects of human development and essential for homeostasis of all organ systems. Much attention has recently been focused on the role of NHRs in energy balance, metabolism, and lipid homeostasis. Dysregulation of NHR function has been implicated in numerous pathologies including cancers, metabolic obesity and syndrome, Type II diabetes mellitus, cardiovascular disease, hyperlipidemia, male and female infertility and other reproductive disorders. This review will discuss the dysregulation of NHR function by environmental endocrine disrupting chemicals (EDCs), and the associated pathological consequences of exposure in numerous tissues and organ systems, as revealed by experimental, clinical, and epidemiological studies.

## 1. Introduction

### 1.1. Nuclear Hormone Receptors: An Overview

The biological actions of steroids, thyroid hormones, retinoids, and other lipophilic hormones are mediated through a family of intracellular receptors termed the nuclear hormone receptor (NHR) superfamily. Most NHRs are ligand-activated transcription factors that reside in the cytoplasm or nucleus. NHRs respond to hormones by associating with transcriptional cofactors, and the receptor–cofactor complexes bind DNA at specific recognition sequences in the regulatory region of target genes. From these sites, the receptors interact with the basic transcriptional apparatus to alter (induce or repress) gene expression. Since NHR cofactors possess chromatin remodeling activities, the association of receptor–cofactor complexes at cognate DNA sites can alter the access and activity of the basal transcription apparatus at the transcription start sites of target genes. The associated changes in gene expression elicited by NHR activation mediate essential developmental, metabolic, and homeostatic functions.

Forty-eight NHRs have been identified in humans. This review will focus on receptors for which ligands have been established, namely Type I (steroid hormone receptors) and Type II (receptors that heterodimer with the retinoid X receptor, RXR). Both Type I and II receptors respond to endogenous ligands by altering gene expression, although the cellular mechanisms utilized by the different receptor classes are distinct. Type I receptors, including the receptors for estrogens (ER-alpha and ER-beta), androgens (AR), progestins (PR), glucocorticoids (GR) and mineralocorticoids (MR), may reside in the cytoplasm or nucleus. These receptors respond to activating ligands by undergoing conformational changes that result in loss of associated heat-shock proteins, homo- or heterodimerization, and translocation to chromatin-containing regions of the nucleus. In contrast, the receptors for thyroid hormones, retinoids, vitamin D, and the peroxisome proliferator-activated receptor subfamily members (PPAR-alpha, PPAR-delta, and PPAR-gamma) are associated with DNA in the absence of ligand together with RXR. In the unliganded form, these receptors are capable of recruiting corepressors and repressing basal target gene transcription.

### 1.2. The Endocrine Disruptor Concept

#### 1.2.1. Endocrine Disrupting Chemicals

The essence of classical NHR action is that endogenous hormones function like a molecular switch, engaging the receptor into a transcriptionally active conformation. However, we know that in addition to native hormones, there exist a wide array of NHR ligands that are obtained from exogenous sources including dietary lipids and vitamins, pharmaceutical agents, plant-derived compounds, and industrial byproducts. These ‘xenochemicals’ may possess a range of agonist, partial agonist or antagonist activities dependent on the dose, presence of endogenous receptor ligand and the nature of the structure–activity relationship (interaction with the NHR). Throughout the 20th and 21st centuries, the frequent use of pharmaceuticals and pesticides in agriculture and the amplification of industrial byproducts have increased concern about the dissemination of chemicals into our environment. The recognition that these chemicals pose human health risks, by means of their ability to interfere with endocrine physiology, led to the classification of these exogenous agents as endocrine disrupting chemicals (EDCs).

The most predominant effects of EDCs to date have described the effect on steroid hormone receptor signaling (estrogen and androgen, thyroid hormone) receptors. Despite the established actions of EDCs mediated through ERs and ARs, the effects of these chemicals on other Type I NHRs such as GR, MR, and PR are not well-described. However, as will also be discussed in this review, there is emerging evidence that interactions of EDCs with other NHRs, notably PPAR-gamma, may coincide with chronic diseases such as obesity and type II diabetes/metabolic syndrome [1,2].

#### 1.2.2. Historical Perspective

The concept of EDCs was introduced in the 1950s when endocrinologist, Roy Hertz, proposed that anabolic steroids fed to livestock could find their way into the human body and mimic endogenous hormone activity [3,4]. This was followed by observations of strange reproductive patterns in wildlife throughout the 1960s, that in retrospect, appeared to be the consequence of dysregulated estrogen and androgen physiology [5].

In the early 1970s, researchers observed a rare vaginal and cervical clear-cell adenocarcinoma in women who were subjected to diethylstilbestrol (DES) in utero. DES, an estrogen-like pharmaceutical thought to prevent miscarriage, had been in clinical use for 30 years. An estimated 5–10 million people in the US were endangered by DES during that timeframe, including pregnant women and the children born of those pregnancies [6]. Subsequent studies reported that in addition to cancer, prenatal DES exposure was associated with anatomical malformations of the cervix, vagina, and uterus, decreased fertility in female offspring, and high incidence of cryptorchidism and testicular hypoplasia in males [7].

The DES medical tragedy introduced the idea that hormone disruption, specifically at certain sensitive periods, could pose a significant threat to human health and development. This realization sparked an intensive study of estrogens and other hormonally-active chemicals in the environment in the decades that followed. To date, there is particularly heightened public awareness and concern over use of food and beverage packaging and other plastics containing the xenoestrogen bisphenol A (BPA) [8].

The consequences of EDC exposure in humans are unclear, yet there are reports of significant data correlating exposure with various diseases, as detailed below. Much of what we do know has been established mainly in the laboratory using cell and animal models. From these studies, it is apparent that contact with EDCs during early development and childhood may cause permanent late stage effects [9,10,11]. Further, EDC contact may produce varying effects in adults, with some adults not showing any significant or visible effects.

#### 1.2.3. The World Health Organization and Endocrine Society Statements on EDCs

In 2012, the World Health Organization collaborated with key scientific experts and the United Nations Environment Program to update the 1st Scientific Statement on Endocrine Disrupting Chemicals [12]. This document, and the previous 2002 report, laid down these foundations:

- The endocrine systems of vertebrates are very similar, and thus, effects shown in animals may also occur in humans.
- Effects on early development are a special concern, as these effects are often irreversible.
- The effects may not become evident until later in life.

In 2015, the Endocrine Society released its 2nd Scientific Report on Endocrine Disrupting Chemicals (12, 16), which made the following declarations:

- Using experimental animal models, causative relationships have been established between EDC exposure and manifestation of disease
- Correlative epidemiological data in humans are consistent with observations made in experimental models
- No safe dose of EDC exposure can be established, as low-dose effects have been observed, and experimental and epidemiological data indicate developmental vulnerability
- EDC exposure is a public health issue, and health care professionals should alert the public about modes of exposure and the need for prevention

This review presents current knowledge concerning the molecular mechanisms of EDCs on NHR signaling as they relate to potential pathological consequences that have been observed in clinical, epidemiological, and experimental studies.

## 2. Endocrine Disrupting Chemicals

### 2.1. Types of EDCs

The Endocrine Disruptor Exchange (TEDX; http://endocrinedisruption.org) lists approximately 1000 heterogenous agents that have been characterized as EDCs.

In general, EDCs can be classified as plant-derived (phytoestrogens), industrial chemicals, manufactured household and consumables, medical items, and pharmaceuticals. A brief description of each class of EDCs is provided as follows:

- **Phytoestrogens** are chemicals that are enriched in flax seed, nuts, soy products, cereals, breads and legumes. Examples are genistein, daidzein and resveratrol, which display estrogenic or antiestrogenic activities via binding estrogen receptors. Phytoestrogens interact with a number of other NHRs as well.
- **Industrial chemicals** that function as EDCs include pesticides, flame retardants, and combustion products. Examples include dioxins, perchlorate, and Dichlorodiphenyltrichloroethane (DDT), the latter of which was banned worldwide in 2001 but still persists in the environment.
- **Common household and consumable items** containing EDCs include cosmetics, toys, food and beverage packaging materials, contaminated foods, and contaminated groundwater. Traditionally, Bisphenol A (BPA) and phthalates have been ubiquitous in household and consumable items, and despite a conscious effort by manufacturers to reduce these chemicals, are still widely used. BPA is starting to be replaced by Bisphenol F (BPF) and Bisphenol S (BPS), and while both of these chemicals are less potent in activation of ERs, BPF shows similar efficacy to BPA in stimulation of ER-alpha transcriptional activity [13].
- **Medical Devices** containing EDCs include intravenous tubing, gloves and plastic devices. The most widespread EDCs present in healthcare and medical devices are BPA and phthalates.
- **Pharmaceuticals** containing natural and/or synthetic steroids can function as EDCs as they may leach into our drinking water and soil. Both DES and natural estrogens are detectable and present at sufficient concentrations to manifest biological effects as demonstrated in animals and postulated in humans [14,15].

### 2.2. EDC Exposure

There are diverse routes of EDC exposure. These include consumption of food, dust, and water, ingestion of breast milk, inhalation of gases and particles in the air, skin contact, or biological transfer across the placenta.

EDCs have been detected in both children and adults in numerous fluids including blood, sweat, urine, breast milk and hair [16,17]. The Center for Disease Control (CDC) has begun monitoring the prevalence and levels of various EDCs as part of an ongoing survey termed the National Health and Nutrition Examination Survey (NHANES). When urinary BPA levels were analyzed as part of the 2013–2014 NHANES, detectable levels of BPA were found in 96% of urine samples taken from approximately 2500 individuals 6 years and older [18]. Phthalates are also known to be ubiquitous; 95–98% of women and children tested positive for phthalates in two NHANES surveys conducted from 1999 to 2014 [19].

Despite the knowledge that EDCs are readily detectable in humans, whether they accumulate at sufficient levels to become physiologically disruptive is controversial. While indeed EDCs elicit adverse effects in many experimental systems (see below), it is unclear whether these findings can be extrapolated to human exposure levels when considering the relatively high doses required to elicit such responses. Some scientists have found adverse effects at low doses in laboratory experiments [20], while others have not been able to corroborate these findings [21]. It is also possible that simultaneous contact with multiple chemicals could provide a summative effect. Many environmental pollutants are found in mixtures, and thus it is likely that a population exposed to an EDC would be subject to multiple agents. Indeed, different classes of EDCs have demonstrated additive or even synergistic effects [22]. Alternatively, many EDCs are lipophilic and are known to bioaccumulate in tissues (e.g., adipose) or body fluids [23]. A recent study showed that following feeding, infants consuming exclusively soy-based formula had serum genistein levels of 1–10 μM [24], which are certainly within the range to elicit physiological effects through estrogen receptors.

## 3. Molecular Mechanisms of EDC Action on Nuclear Hormone Receptors

EDCs utilize numerous mechanisms to interfere with NHR physiology. EDCs can mimic or antagonize the effects of native hormones, stimulate or inhibit the production and metabolism of endogenous hormones, or disrupt peripheral transport of hormones to their target tissues [23]. There are many potential activities of EDCs, but we currently know most about their estrogenic and anti-androgenic activities that are mediated through binding estrogen and androgen receptors.

### 3.1. Classical Nuclear Hormone Receptor Pathway

The ability of EDCs to activate or inhibit NHR function is the most understood mechanism by which EDCs mediate their cellular effects. As discussed above, the classical pathway of NHR action involves ligand binding, receptor association with transcriptional cofactors, DNA binding to regulatory elements in different target genes, and modulation of transcriptional activity to cause differential gene expression. EDCs are known to participate in all of these aspects of classical NHR action.

#### 3.1.1. Ligand Binding

EDCs are known to occupy the ligand-binding pockets of numerous NHRs [25]. In general, however many EDCs bind with significantly (≥ 1000-fold) lower affinities compared with endogenous hormones [26,27]. The relatively low binding affinities of EDCs, and high doses required for activation [28], raises the question of whether these agents manifest their deleterious effects through classical NHR signaling pathways or alternative pathways as described below.

#### 3.1.2. Agonist/Antagonist Activity

Foundational work in the NHR field yielded the principle that different ligands induce different conformational changes in NHRs, resulting in unique biological activities [29,30]. EDCs are known to induce diverse conformational changes in NHRs as well as possess a range of different activities: agonist, partial agonist, or antagonist. It is also not uncommon for a ligand to display receptor-selective actions. For example, BPA is a partial agonist of PPAR-gamma and estrogen receptors, but a competitive antagonist of ARs [31,32].

#### 3.1.3. Cofactors

As discussed above, NHRs engage transcriptional cofactors as a consequence of conformational changes induced by ligand binding. A major mechanism for ligand-specific biology lies in differential cofactor recruitment [33]. EDCs are known to differentially associate with transcriptional coactivators compared with estradiol or other ER-alpha and ER-beta ligands [34,35], and thus, the degree of agonist/antagonist activity of specific EDCs on a particular receptor may in part reflect the degree of cofactor association. No EDC-specific cofactors have been identified for NHRs, rather, it is believed that EDCs engage existing cofactors in a ligand- and tissue-selective manner, as do the various natural and synthetic ligands for NHRs.

#### 3.1.4. DNA Binding and Differential Gene Expression

In addition to ligand, DNA is known to be an allosteric modulation of NHR function [36]. ERs bound to estradiol or various EDCs displayed unique preferences for cofactors when situated on the different DNA response elements selected from 4 ER target genes [35].

Furthermore, like estradiol and other ER ligands, EDCs display different activities on distinct NHR target genes, suggesting that the structure and function of the EDC–receptor–cofactor complex is influenced by the promoter complex [28].

### 3.2. Nongenomic Nuclear Hormone Receptor Signaling

In addition to established effects on gene expression, some NHR ligands are known to mediate nongenomic signaling through membrane-associated hormone receptors. The most well described are the nongenomic effects of estrogens, which are mediated through G protein-coupled estrogen receptor 1 (GPER1/GPR30), a Gs-coupled heptahelix receptor. Activation of GPER1 by estradiol results in stimulation of adenylyl cyclase, the release of membrane-tethered epidermal growth factor (EGF), and a cascade of intracellular events that result in hormone responses which are rapid compared with those occurring from classical genomic NHR signaling [37].

The development of GPER1-selective ligands, together with GPER1 knockout animals, has revealed unique physiological roles of this receptor compared with the classical nuclear ERs. GPER1 deletion results in altered poor glycemic control, insulin resistance, and hypertension [37]. Accordingly, GPER1 agonists, stimulate vasodilation in the vasculature [38], and enhances insulin release from pancreatic beta cells [39], further suggesting a role for the receptor in mediating the anti-diabetic and anti-hypertensive actions of estrogens. GPER1 agonists also prevent inflammation and degeneration of dopaminergic neurons in an animal model of Parkinson’s disease [40]. Analysis of GPER1 expression in 361 human breast carcinomas revealed that unlike nuclear ERs, GPER1 expression showed a positive association with HER-2/neu status, tumor size, and metastasis [41]. It is likely that the receptor mediates the physiological and pathological roles of estrogens in numerous tissues, and thus, the association of GPER1 with multiple disease states has prompted interest in the receptors as a therapeutic target.

GPER1 demonstrates specificity for estrogens compared with other steroid hormones. However, recent studies show that several EDCs bind the receptor with considerable affinity compared to estradiol, and elicit estrogen-like responses [42,43,44]. In addition, there is emerging evidence that EDC signaling though GPER1 may have pathological consequences. Two recent studies demonstrated that low dose phthalates activate GPER1, resulting in increased proliferation of breast cancer cells [45] and cervical cancer cells [46]. Comparison of the relatively low affinity of EDCs for genomic ERs to the relatively high affinity for GPER1 has led to the hypothesis that at least some of the estrogenic effects of EDCs could be mediated through non-genomic ER-alpha or ER-beta signaling rather than the classical pathway [47].

### 3.3. Epigenetic Effects

Emerging evidence suggests that early life exposure to EDCs during critical developmental periods may result in reprogramming of normal biological responses in a manner that predisposes one to diseases in adulthood. In the DES tragedy described above, the offspring of pregnant women taking DES developed significant reproductive abnormalities and cancers later in life as a result of in utero exposure [7].

It was previously believed that gene–environment interactions may impact disease susceptibility by inducing genetic mutations. In contrast, ‘developmental reprogramming,’ a new type of gene–environment interaction, refers to epigenetic modifications made on the genome during the pre-natal and peri-natal periods. These modifications involve methylation of cytosine and guanine-rich stretches of DNA (CpG islands), and histone methylation, both of which can create alterations in gene expression [48]. It should be noted that DNA methylation is an essential aspect of normal development, however, dysregulated methylation induced by environmental exposures is known to contribute to disease. Indeed, it is now believed that exposure to environmental stressors during development may predispose one to cancer in adulthood, not by inducing genetic mutations as long believed, but rather, by inducing epigenetic changes [49].

The use of experimental models has demonstrated that a number of EDCs induce epigenetic reprogramming [48]. Recent studies implicate this as a possible mechanism for the rise of obesity and insulin resistance [50]. In utero exposure of mice to BPA induced promotor hypermethylation and decreased expression of *Gck*, a diabetes susceptibility gene that is essential for glucose metabolism in hepatocytes. Strikingly, these animals displayed glucose and insulin intolerance and were able to transmit the genotypic and phenotypic alterations induced by BPA exposure to their offspring [51]. The transgenerational effects of chemical exposure and link to obesity was observed in animals with prenatal exposure to the marine paint additive Tributyltin (TBT). The treated animals and subsequent two generations of offspring displayed increased adipocyte number and size, and enlarged adipose depot, despite a normal caloric consumption [52]. The convergence between obesity, epigenetic modifications, and NHR function was observed in a recent review that linked EDC exposure to altered gene methylation and increased functional activity of PPAR-gamma, the master regulator of adipogenesis. Importantly, the epigenetic modifications in the PPAR-gamma gene were associated with increased adiposity, which supported the premise that EDCs mediate their obesogenic effects through alteration of the methylation status and subsequent expression of PPAR-gamma [53].

Nongenomic NHR signaling is also a means by which EDCs may mediate changes in the epigenome. Activation of membrane ERs by EDCs and induction of PI3K/AKT signaling resulted in alterations of methyltransferase activities. While the downstream biological consequences are not yet defined, it appears that EDCs may impact the epigenome by inducing NHR pathways that alter the activities of the cellular epigenetic machinery [54].

## 4. EDCs and Reproductive Disorders

EDCs alter the function of neuroendocrine regulatory systems, and the most well-studied effects are on the hypothalamic-pituitary-gonadal (HPG) reproductive axis. Various aspects of EDC alteration of HPG signaling will be described below with regards to precocious puberty, female and male reproductive function, and hormone-related cancers.

### 4.1. Precocious Puberty

#### 4.1.1. Observed Effects of EDCs

The initiation of puberty and subsequent physiological and anatomical changes occur as a consequence of activation of the hypothalamic-pituitary-gonadal (HPG) axis. In the 1960s, the normal age range of pubertal onset was designated as between 8 and 13 years in girls and between 9.5 and 13.5 years in boys. More recently, cross-sectional data revealed that pubertal milestones were being reached earlier in the US, and these observations led to recommendations to classify pubertal development as precocious when it occurs before the ages of 8 years in girls and 9.5 years in boys [55].

There has been wide speculation that the rise in early puberty may be partially caused by environmental factors [56]. Indeed, there is both experimental and correlative clinical evidence that EDC exposure may alter pubertal timing. Pre-mature thelarche and pubic hair were observed in girls between 14 months and 8 years of age following the use of hair products containing estrogens or placenta extracts [57]. Pseudo-precocious puberty was reported in seven infants following regular topical use of an estrogen-containing lotion. This was manifest by intense pigmentation of the mammillary areola, linea alba of the abdomen and genitals, mammary enlargement, and presence of pubic hair [58]. It is also clear that the association of environmental chemicals with early pubertal changes is not limited to chemicals within topical healthcare products. Higher serum levels of phthalates were present in girls from Puerto Rico who displayed premature breast development [59]. Furthermore, sexual precocity in immigrant girls in Belgium was associated with high plasma levels of p,p’-DDE, a metabolite of the organochloride pesticide DDT [60]. DDT use was banned in the US in 1972, however, it remains readily detectable in US soil to date [61]. Despite the risks and possible consequences of exposure, DDT is still widely used in Mexico, South America, Africa, and Asia.

Clinical data also suggest that EDC exposure during the prenatal and perinatal periods may accelerate age of puberty. Following a food contamination accident in the 1970s, more than 4000 individuals consumed meat and dairy products containing polybrominated biphenyls (PBBs), a component of fire retardants. Girls who were subject to high levels of PBBs in utero and in some, through breastfeeding, achieved menarche at approximately 1 year earlier than those with low exposure or girls who were not breastfed [62].

There also exists evidence that EDCs may alter pubertal timing in males. Two studies have associated topical application of hair or dermal creams containing estrogenic chemicals with development of gynecomastia in pre-pubertal boys [63,64,65].

#### 4.1.2. Mechanism of EDCs

Central precocious puberty may occur as result of early activation of pulsatile gonadotropin-releasing hormone (GnRH) secretion, and the cause is usually unknown. However, a causal link between EDCs and central precocious puberty was established in experiments in which early postnatal exposure of female rodents to estradiol or DTT was followed by early maturation of pulsatile GnRH secretion [66]. Thus, early initiation of HPG activity and puberty may occur in pre-pubertal individuals with transient exposure to exogenous estrogens [67]. GnRH secretion and the onset of puberty are regulated by the hypothalamic peptide hormone, kisspeptin. Estradiol and several EDCs with estrogenic activities were shown to decrease GnRH activation by interfering with the kisspeptin system in the hypothalamus [68], suggesting a potential mechanism by which EDCs accelerate onset of puberty in exposed individuals.

Peripheral precocious puberty refers to early pubertal development as a consequence of inappropriate exposure to estradiol, testosterone, or chemicals with estrogenic/androgenic activities. Clinical studies have described peripheral precocious puberty in preadolescent males who developed gynecomastia and premature thelarche in females following topical administration of products containing lavender or tea tree oils [65,69]. Mechanistic analysis revealed that the oils and their constituents activated ERs in human breast epithelial cells and increased ER-alpha-dependent gene transcription and cell proliferation. Lavender and tea tree oil were also designated as EDCs via their ability to antagonize AR function. It was concluded that the repeated exposure to the estrogenic and anti-androgenic components of the oils was likely responsible for the prepubertal gynecomastia in the boys [65,69].

### 4.2. Female Reproductive Development and Function

#### 4.2.1. Female Reproductive Development

Early life exposure to EDCs alters development of the female reproductive system, and current data suggest that EDCs may pose the greatest risk during prenatal and early postnatal development when organ and neural systems are forming [70]. This is certainly exemplified by the observed effects of in utero exposure to DES. Prenatal or neonatal treatment of rodents with DES resulted in structural abnormalities affecting the uterus, cervix, fallopian tubes, vagina and ovaries in females and alterations in exposed male offspring [71,72]. In humans, the DES medical tragedy revealed that human contact with DES during prenatal development also resulted in adverse reproductive system phenotypes. In light of these observations, it is concerning that high levels of multiple EDCs are found in human amniotic fluid and umbilical cord blood [73].

Early exposure to BPA also remains a significant concern. BPA was a component of baby bottles, pacifiers, baby food packaging, and infant and children’s toys until its banning by regulatory agencies in 2008 [74]. Experimental studies in adult female rodents have linked exposure to BPA levels (similar to those experienced by humans) to the development of a number of reproductive pathologies including ovarian cysts, cervical sarcoma, uterine polyps, and mammary adenocarcinoma [75].

#### 4.2.2. Ovarian Function and Fertility

A key aspect event in female reproductive development and function is folliculogenesis, the maturation of ovarian follicles. This process allows for ovulation of the oocyte for subsequent fertilization. Follicles consist of the oocyte and somatic cells (granulosa and theca cells) that produce sex steroid hormones. The process of folliculogenesis involves multiple stages. During fetal development, primordial follicles become primary follicles that arrest until puberty, at which time they may mature into antral follicles prior to ovulation.

EDCs can interfere with folliculogenesis at numerous stages of the process. A decrease in the population of primordial follicles and altered development of primary, preantral, or antral populations of follicles, or corpus lutea has been observed following exposure to BPA, phthalates, pesticides, or airborne pollutants [76,77,78,79,80]. In a study of 243 women undergoing IVF, the presence of nicotine metabolites in follicular fluid was correlated with a 50% reduction in the production of mature oocytes in women >40 years old [81]. It was suggested that this may be one reason that the negative effects of smoking become clinically detectable in older women. Importantly, we now know that a number of diverse chemical agents can destroy follicles prematurely, and thereby hasten entrance into the menopausal state [76].

EDCs may also alter the processes of ovulation and fertilization in humans. In correlative studies, higher levels of PCBs (industrial EDCs persistent in our environment) were found in anovulatory females between 21 and 38 years [82]. Furthermore, levels of the pesticide DDE were associated with failed fertilization in women undergoing IVF [83].

Approximately 10–25% of fertilized human oocytes are aneuploid, and thus, aberrations in chromosomal number are the leading cause of miscarriage or congenital defects [84]. Smoking is known to alter the meiotic spindle of human oocytes in a manner that leads to chromosome errors and disruption of reproductive outcomes [85]. There are also reports that correlate maternal EDC intake with risk of congenital abnormalities. One well-publicized study involves a cohort of mothers in a Hungarian village who consumed fish contaminated with high levels of the insecticide, trichlorfon, during pregnancy. Of the 15 live births by these women in 1989–1990, 11 (73%) of offspring were affected by birth defects, including down syndrome [86].

Experimental studies have also linked exposure to EDCs during pregnancy to decreased rates of implantation, elevated rate of miscarriage, and decreased viability of neonates [87]. Female rodents subjected to the phthalate DEHP experienced reduced rates of implantations and increased fetal resorption. Pups born to these mothers had low birth weights, and possessed anatomical malformations [88]. In clinical studies of EDC exposure, it was observed that while most DES daughters were able to carry a child to term, in those with anatomical alterations of the reproductive tract, there was only a one-third chance of a successful pregnancy [89]. Other studies of chemical contact on human reproductive outcomes are more ambivalent. It has been suggested that the equivocal findings in human studies may be the consequence of small sample size, poor methodology and multiple confounding factors [90].

There is emerging evidence that EDCs have significant effects on the production and circulation of reproductive hormones, including steroid hormones, as well as gonadotropins, luteinizing hormone (LH) and follicle-stimulating hormone (FSH). Kisspeptin is a hypothalamic hormone that regulates release of GnRH, LH, and FSH, and the ovulatory LH surge [91], and numerous EDCs are known to interfere with kisspeptin signaling in the hypothalamus [68]. In addition to hormone release, several EDCs have the potential to alter steroidogenesis [92,93].

#### 4.2.3. Polycystic Ovarian Disease

Polycystic ovarian disease (PCOS) is a common disorder among women of reproductive age that is characterized by enlarged ovaries containing small collections of fluid. Women with PCOS frequently experience elevated androgens, infrequent or prolonged menstrual periods, excess hair growth, acne, and metabolic complications, including insulin resistance and obesity [94]. While the etiology is complex, there is a notable association of PCOS with exposure to exogenous androgens [95]. Nicotine possesses androgenic activity, and women smokers with PCOS demonstrate elevated testosterone at levels sufficient to exacerbate existing insulin resistance [96]. BPA concentrations are elevated in women with PCOS compared with reproductively healthy women [97,98,99], and in the same women, BPA levels positively correlate with degree of hyperandrogenemia [98,99], which causes many of the PCOS symptoms. Although a causal relationship has yet to be established, the human studies are backed by experimental observations, where prenatal exposure to EDCs increases the incidence of polycystic ovaries in rodent offspring, presumably by interfering with estradiol-dependent normal follicular development [100].

#### 4.2.4. Endometriosis

Endometriosis, growth of uterine tissue in other portions of the reproductive tract, is a gynecological condition associated with inflammation, pain, bleeding, and sometimes infertility. Stimulation of ectopic uterine growth is estrogen-dependent, and thus, it has been suggested that EDC exposure could be associated. The Endocrine Society has concluded that thus far, further studies are required to establish a causative association between EDCs and risk of endometriosis [48].

There is, however, strong evidence for a role of dioxin exposure in the etiology of endometriosis. Dioxin has estrogenic activities and the agent stimulates growth of human uterine epithelium cells in vitro [101]. A series of human and non-human primate studies have also linked exposure to incidence and pathogenesis of endometriosis [102]. Progesterone action is crucial to decreasing inflammation in the normal endometrium, however, in endometriosis, progesterone resistance results in a proinflammatory environment that promotes ectopic endometrial growth [103]. A number of studies have linked dioxin exposure to abnormal endometrial growth, increased estradiol biosynthesis, and upregulation of inflammatory chemokines, which results in an inflammatory environment amenable to the growth of endometrial lesions [104,105,106].

#### 4.2.5. Uterine Fibroids

Uterine fibroids, benign tumors of the myometrium, affect 70–80% of females. Women clinical presentations include bleeding and pain, leading to infertility, miscarriage, and other reproductive disorders [107]. Clinical data have revealed that fibroids require estrogen and progesterone receptor activity, and both ovarian hormones stimulate fibroid growth during the female reproductive years [108].

Epidemiological data highlight EDC exposure in the pathogenesis of uterine fibroids. Specifically, the Nurses’ Health Study II, revealed that prenatal contact with DES increased risk for fibroids by 13% in women aged over 35 years [109]. Additional positive correlative data were provided by The NIEHS Uterine Fibroid Study of 1364 DES-exposed or unexposed women aged 35 to 49 years. When screened for uterine fibroids, the odds ratio for large tumors was 2.4 for Caucasian women subjected to DES in utero [110]. Experimental studies have shown that in utero exposure to DES or genistein results in changes in the histone methylation patterns of estrogen-responsive genes in a manner that causes hyper-sensitivity to hormones. These epigenetic modifications were shown to be causative in accelerating the formation of fibroids in the myometrium of adults with the early exposures [111].

### 4.3. Male Reproductive Development and Function

What do (1) altered fetal reproductive development, (2) decreased semen quality, and (3) testicular germ cell cancer (TGCC) have in common? In 2001, Skakkeback and colleagues coined this triad of abnormalities ‘Testicular Dysgenesis Syndrome’ (TDS), and proposed that the 3 maladies share a common etiology by which environmental chemicals and genetics result in abnormal development of the fetal testis [112]. A discussion of the role of EDCs in TDS follows below.

#### 4.3.1. Male Reproductive Development

Male wildlife subjected to EDCs display a number of reproductive developmental abnormalities, including hypospadias, cryptorchidism, low sperm counts, and decreased penis size [113,114,115]. The incidence of similar deformities is currently rising in the human population [116]. Adverse effects of EDCs on human male reproductive development were seen as a consequence of the DES tragedy. In two independent studies of DES-sons, in utero exposure to DES was linked to a significant increase in the incidence of epididymal cysts and hypoplastic testes, cryptorchidism, and testicular inflammation [117,118]. Seminal vesicle atrophy following developmental exposure to DES has also been observed, and these effects were shown to result from ER-alpha-dependent changes in DNA methylation [119]. Case control studies have correlated the increased incidence of congenital cryptorchidism with the levels of pesticides found in maternal breast milk samples [120]. However, other epidemiological studies reported little or no correlations between EDC exposure and male reproductive developmental malformations [121,122]. While it is widely acknowledged that both EDC contact and male reproductive disorders are on the rise, there is a deficit of studies and analysis examining their relationship with regards to clinical developmental abnormalities.

#### 4.3.2. Semen Quality

Epidemiological and clinical data have continued to build a strong relationship between EDC exposure and the decline in human sperm quality. Various aspects of testicular integrity, and spermatogenesis are androgen-dependent, and the ability of EDCs to disrupt androgen signaling in the testes is linked to the effects of numerous EDCs on semen quality. Sufficient information about effects related to specific EDCs is available as follows:

- **DDT:** DDT is known to adversely affect semen quality, particularly in malaria-endemic regions where the pesticide has been sprayed regularly. Specifically, high serum DDT has been associated with decreased sperm motility, morphology, count, and semen volume in young Mexican and South African males [123,124].
- **Vinclozolin:** The effects of other EDCs on semen quality have been investigated in both experimental and clinical settings. The fungicide, vinclozolin, functions as an androgen antagonist in animals, and when administered to pregnant rodents resulted in male offspring with feminized reproductive tracts, low sperm count, and other deformities [125]. These consequences of exposure, however, have yet to be reported in humans, as no anti-androgenic effects were found in a study of men with occupational exposure to the agent over a period of 1–13 years [126].
- **BPA:** Several recent epidemiological studies have correlated BPA exposure with a decline in multiple characteristics of sperm fitness [127,128,129]. Interesting, the reported serum BPA concentrations in the men in these studies were within the range known to activate GPER1, the membrane ER that mediates nongenomic effects of estrogens [44].
- **Phthalates:** A series of reports have described the anti-androgenic activity of several phthalates, suggesting that these agents may affect fertility. Exposure of humans to phthalates is a universal occurrence, and the majority of humans contain detectable levels in body fluids [19]. Rodent studies have shown that treatment of pubertal and adult animals with phthalates can impair spermatogenesis and cause testicular atrophy [130]. However, available human adult data suggest that exposure to phthalates at environmental levels is unlikely to have significant effects on parameters of sperm fitness [131].

### 4.4. Hormone-Related Cancers in Males and Females

#### 4.4.1. Testicular Cancer

Testicular cancer is the most common cancer arising in young men, and 95% of cases develop from testicular germ cells (TGCC). A steep rise in the incidence of testicular germ cell cancer (TGCC) over the past few decades has been observed [132,133,134]. It has been speculated that environmental exposures may be contributory, however available evidence is conflicting. A recent clinical report proposed that male serum DDE levels may be positively associated with the risk of both seminomatous and nonseminomatous TGCCs [135]. However, in case-controlled studies, no association was made between serum PCBs, DDE, or benzenes, and risk of any type of TGCC [136,137]. In review of all the currently available human data, it is apparent that while the incidence of TGCC has increased over the past 40 years, the reasons for this rise are still unclear [138].

#### 4.4.2. Breast Cancer

Most studies on the carcinogenic effect of EDCs in females are centered on the mammary gland and uterus, as these are the main estrogen target organs in females. Data on the role of EDCs in uterine hyperplasia and cancer are very limited to date, so the discussion below focuses primarily on EDCs and breast cancer.

Lifetime exposure to ovarian hormones is an established risk factor for breast cancer [139]. Accordingly, there exists significant concern on the long-term effects of EDCs with estrogenic activities on susceptibility of the breast during development and in adulthood. Experimental reports have implied that early contact with xenoestrogens may result in altered morphology of the mammary gland, which could potentially prompt carcinogenic changes [140]. Epidemiological studies have also suggested that hormone exposure in the intrauterine environment may affect the risk of developing breast cancer later in life, as excess estrogen exposure in utero has been associated with elevated breast cancer incidence [141]. Additional evidence for a prenatal developmental window of exposure is seen among the DES daughters, as those age 40 years and older display a 2.1 greater incidence of breast cancer than aged-matched unexposed subjects [142]. Likewise, women subjected to the estrogenic pesticide DDT in utero are reported to also possess a significant increase in risk of developing breast cancer later in life [143].

The effect of EDCs on the breast during post-natal development and in the adult are not clear. Girls with high serum levels of the pesticide DDT around the age of puberty were five times more likely to develop breast cancer later in life compared with unexposed peers [144], and one study linked total EDC contact in adults to incidence of breast cancer [145]. On the other hand, two independent studies failed to show a difference in serum BPA levels in adult breast cancer patients vs. disease-free individuals [146,147]. There is also debate whether members of the phytoestrogen class of EDCs have tumorigenic or protective effects in the breast. Phytoestrogens such as genistein and resveratrol have shown chemopreventive effects in animal models [148], and epidemiological studies link childhood soy consumption with decreased incidence of breast cancer later in life [149]. Additional epidemiological studies evaluating the effects of phytoestrogens alone, and in combination with other estrogenic chemicals, will be required to properly assess safety. However, the knowledge that chronic estrogen intake is linked to increased risk of developing breast cancer later in life has prompted current studies of phytoestrogen consumption in infants, which occurs through ingestion of soy-based formulas [150].

The mechanisms of potential EDC-mediated carcinogenicity are not clear. Numerous EDCs are known to alter estrogen-regulated gene expression in human breast cancer cells [151,152] as well as enhance local estradiol synthesis and breast cancer cell proliferation [153]. EDCs may also have the potential to induce cancer metastasis through regulating markers of the epithelial-mesenchymal transition and cellular migration pathways [154].

Recent studies highlight the role of altered cell-to-cell and cell-to-extracellular matrix communications in the development of tissue malignancy, a concept known as tissue organization field theory (TOFT) [155]. Breast tissue development involves reciprocal interactions between the epithelium and stroma, and the breast cancer stromal microenvironment has a critical role in promoting tumor progression [156]. Experimental data suggest that EDCs promote breast cancer progression through modifying inter- and intra-cellular signaling in the tumor stroma in a manner that stimulates growth and invasiveness of epithelial cells [157].

#### 4.4.3. Endometrial Cancer

There is currently very little human data relating EDCs to endometrial cancer. A recent meta-analysis of available human and experimental statistics from 1992 to 2016 showed a potential carcinogenic effect of EDCs in the endometrium, although it was concluded that additional epidemiological studies are needed before any steadfast conclusions can be made about human risk [158].

#### 4.4.4. Prostate Cancer

An estimated 1 in 7 men living in the US will develop prostate cancer during their lifetime [159]. Despite the high incidence among the general population, epidemiological studies have revealed a highly significant elevation of prostate cancer in individuals chronically exposed to pesticides as a consequence of their agricultural occupations [160,161]. Additional EDCs, including arsenic, cadmium, and PCBs have also been linked to prostate cancer [12].

Although it remains to be determined how various EDCs affect prostate carcinogenesis, the estrogenic properties of these agents are thought to be contributory. A strong association exists between chronically high-level estrogens in men and risk of prostate cancer [162]. In utero exposure to estrogens, via maternal oral contraceptive use during pregnancy, is associated with increased prostate cancer incidence and mortality in male offspring [163].

EDC exposure also causes epigenetic modification and changes in expression of several genes involved in prostate cancer initiation and growth [164,165]. These changes include increased methylation and decreased gene expression, which was notable, given that expression of these same genes was correlated with recurrence-free survival in prostate cancer patients [164].

## 5. Effects of EDCS in Non-Reproductive Tissues

Given the broad expression profile of NHRs, it is not surprising that EDCs can disrupt function in a wide range of organ systems and tissues. The observed rise in obesity, insulin resistance, metabolic syndrome, cardiovascular disease, and neurocognitive defects parallels the trends in increased human EDCs exposure, as discussed below.

### 5.1. Obesity and Insulin Resistance

Obesity is a severe medical issue, that is widespread both nationally and globally, where it promotes cardiovascular disease, cerebrovascular incidents, Type 2 diabetes mellitus, and other leading causes of death. According to the Centers for Disease Control (CDC), to date 68% of US adults are overweight and 36.5% qualify as obese [166]. There are recent epidemiological studies that demonstrate a strong, positive correlation between EDC exposure, body fat content and obesity, which has led to the term “chemical obesogens” in reference to pro-adipogenic EDCs [167]. An expert panel in the European Union evaluated evidence for probability of causation by examining exposure-response relationships for several common EDCs. From this analysis, it was concluded that there existed a 40% to 69% probability of phthalate exposure causing 53900 cases of obesity in older women [168]. Using the 1999–2002 data from the CDC National Health and Nutrition Examination Survey (NHANES), a positive correlation was made between urinary concentration of four phthalate metabolites and weight circumference in adult males [169]. BPA is also being closely investigated as a potential obesogen. A meta-analysis of epidemiological data found a positive relationship between urinary BPA concentration and obesity in 6 out of 7 studies, and BPA and elevated waist circumference in 5 out of 5 studies [170]. Experimental studies have also shown that in utero or neonatal exposure to BPA is associated with higher body weight [171]. From the existing epidemiological and experimental studies has emerged the “developmental obesogen” hypothesis, which proposes that chemical exposures may predispose one to obesity by altering the differentiation of adipocytes or interfering with development of neurohormonal pathways that regulate feeding behavior. Indeed, experimental studies have shown that treatment with BPA or other EDCs may cause epigenetic changes within adipogenic genes [53,172,173,174].

Together with obesity, the incidence of diabetes continues to rise by epidemic proportions, which has also prompted interest in potential environmental influences. Indeed, multiple recent studies link BPA to insulin resistance. A cross-sectional analysis of 300 women of reproductive age showed that high urinary BPA was associated with insulin resistance and adiposity [175]. In a study of pregnant women, urinary BPA concentrations were positively correlated with elevated blood glucose levels during the second trimester [176]. Furthermore, women of all ages who possess elevated levels of urinary phthalates show an increased incidence of diabetes mellitus [177].

### 5.2. Childhood Obesity

Statistics indicate that an alarming 15–20% of children and adolescents living in the US are obese [178]. This observation has prompted interest into potential developmental effects of EDCs on metabolism and feeding. In animal models, perinatal exposure to low-dose BPA was shown to alter hypothalamic regulation of feeding. Specifically, offspring were resistant to normal physiological leptin-mediated suppression of food intake. Offspring also were lacking pro-opiomelanocortin (POMC) projections into the paraventricular nucleus, indicative of dysfunction in the hypothalamic melanocortin hormones and receptors that control appetite and feeding behaviors (melanocortin system) [179]. Estrogen, an anorectic hormone, is known to increase the number and activity of POMC circuits in the feeding centers of the hypothalamus. Thus, it is possible BPA may alter appetite control by disrupting normal estrogen and ER signaling and interactions with the melanocortin system.

Epidemiological data on EDCs and childhood obesity have been emerging as well, particularly regarding potential effects of BPA and phthalates. The European Union ‘Study of Probability of Cause’ reported that prenatal BPA exposure had a 20% to 69% probability of causing 42,400 cases of childhood obesity [168]. The NHANES 2003–2006 data of 1860 US children aged 8–19 years also indicated a positive link between BPA and adiposity. Of particular interest were the significant gender differences observed. Urinary BPA levels were positively associated with elevated fat mass index in girls, yet correlated with elevated lean body mass index in boys [180]. Whether these gender differences reflect variations in the mechanisms by which BPA alters endogenous hormone signaling remains to be determined. Prenatal exposure to phthalates was also associated with decreased BMI in boys, but with either no change or increased in BMI in girls [181,182]. A recent report, however, described a positive association between phthalate contact in utero and obesity in children 5–12 years of age [183]. There exist several other studies examining BPA and phthalates and childhood obesity, however the associations have been inconsistent from study-to-study [9].

More consistent data have emerged suggesting that prenatal exposure to perfluoroalkyl substances (PFAS) may affect fetal growth and subsequent risk of childhood obesity. PFAS are chemicals used for waterproofing clothing and shoes, and for the nonstick features of some cookware and food packaging. PFAS are a family of chemicals, and the four most commonly found in humans are perfluorooctanoic acid (PFOA), perfluorooctane sulfonate (PFOS), perfluorononanoic acid (PFNA) and perfluorohexane sulfonate (PFHxS). Each of these agents has been detected almost universally in the serum of pregnant women, neonates and children [184,185,186]. In a meta-analysis of 18 epidemiological studies, prenatal PFOA exposure was associated with a 19 g decrease in birth weight [187], a result consistent with that seen previously in 21 animal experiments [188]. It has been suggested that the PFOA-mediated decline in fetal growth might elevate the risk of obesity and metabolic disorders, given that fetal growth deceleration is associated with increased adiposity in childhood [189,190]. Indeed, several studies have shown that prenatal contact with PFOA gives rise to increased adiposity during childhood and adulthood [191,192,193]. Studies of childhood exposure, using the 2003–2004 NHANES data, revealed PFAS levels tracked positively with total and non-high-density cholesterol, although no relationship with body size was observed [194].

In summary, clinical data suggest a positive association between exposure to BPA, PFAS, and perhaps other EDCs, with child and adulthood adiposity. Due to the existence of conflicting results between some studies, additional information including mechanistic data will be required before making firm conclusions about the obesogenic effects of EDCs in humans. Regardless, with the world-wide issue of obesity and metabolic syndrome, an understanding of the role of EDCs in obesity is essential to risk-assessment of exposure to environmental toxins for populations in highly developed economies, both in the US and globally.

### 5.3. Cardiovascular Disease

Cardiovascular disease (CVD) is the leading cause of premature death in the world, and it is widely acknowledged that environmental factors contribute to CVD risk, incidence, and severity. Residents of highly polluted areas display higher levels of cardiovascular risk, and harmful effects have been noted even at exposure levels below those which meet current regulatory standards [195]. The effects of airborne pollutants such as cigarette smoke and exhaust fumes have been well studied. The primary clinical outcomes of chronic contact with urban air pollution are hypertension, ischemic heart disease, and heart failure [195]. Even short-term contact with fine particulate air pollution is associated with increased hospital admissions for cardiovascular disease [196,197]. Short-term exposures in particular are associated with acute ischemic events, suggesting that inhaled toxins may exert their toxic effects within minutes-to-hours [195].

BPA exposure during both childhood and adulthood has been linked to incidence of CVD. Children who experienced in utero exposure to elevated BPA levels possessed significantly higher diastolic blood pressure at age 4 [198]. In adults, the association of BPA with CVD was first reported during review of the 2003–2004 NHANES data, where in 1455 adults, the mean urinary BPA levels were higher in those diagnosed with coronary artery heart disease, angina, and myocardial infarction [199]. Likewise, BPA exposure was linked to risk of coronary artery disease and hypertension in a meta-analysis of 33 epidemiological studies [170]. The likely mechanism involves the ability of BPA to disrupt normal estrogen-ER signaling in the vasculature, which normally affords vascular protection by promoting vascular injury repair and upregulation of vasodilators such as nitric oxide. Finally, it has recently been suggested that serum BPA levels could be used to predict the progression of chronic kidney disease in hypertensive individuals [200].

Not all EDCs are associated with CVD. In fact, there is evidence suggesting that phytoestrogens can exert cardioprotective effects. Indeed, populations consuming large quantities of phytoestrogens show decreased incidence in cardiovascular disease [201]. Epidemiological studies have also shown that in individuals with a high risk of CVD, an increased intake of soy and legumes was associated with lower degree of carotid atherosclerotic burden and better vascular endothelial function [202]. A meta-analysis of 29 published studies found that the substitution of soy protein for animal protein significantly decreased serum concentrations of total cholesterol, low-density lipoprotein (LDL) cholesterol, and triglycerides [203]. This was attributed to the ability of soy to activate the ERs and mimic the long-term antilipidemic effects of estrogen. The use of phytoestrogens in menopausal women as an alternative to traditional hormone replacement therapy has been proposed, although further evaluation of clinical safety would be mandated before consideration of widespread use [204,205,206].

### 5.4. Kidney Disease

Chronic Kidney Disease (CKD), as defined by the National Institute of Diabetes and Digestive Kidney Disease (NIDDK), is any condition resulting in slow-onset kidney decline, leading to increased urine albumin excretion, decreased glomerular filtration rate (GFR), or both [207]. According to the Center for Disease Control (CDC), 15%—or approximately 37 million people—of the US population have CKD, with many cases progressing to irreversible end-stage renal disease (ESRD) [208]. Certain comorbidities, specifically diabetes and hypertension, are currently considered the major predisposing risk factors for CDK development. However, environmental exposures, including EDCs, are generating more attention in studies aimed at understanding the molecular pathophysiological changes associated with CKD.

Since CKD often begins with an asymptomatic period, it is hypothesized that prior EDC exposures can unknowingly inflict damage to various structures in the kidney. Past studies have correlated kidney dysfunction in children with increased exposure to documented EDCs, including phthalate DEHP and BPA [209,210,211]. In both cases, structural changes within the glomerulus resulted in micro-albuminuria, decreased glomerular filtration rates (eGFR), and elevated albumin-to-creatinine ratios (ACR).

In adults, data from the 1999–2000 and 2003–2008 NHANES studies demonstrated that serum PFAS were positively correlated with CKD [212]. Furthermore, urinalysis has revealed evidence of EDCs as risk factors for CDK. A cohort study examining kidney function via the albumin-to-creatinine ratio (ACR), found a positive association between ACR and the urinary EDC metabolites, monobutyl phthalate and benzophenone-1 [209].

A significant area of concern focuses on the secondary health consequences resulting from impaired renal filtration and absorption. This has been shown in patients with renal failure, who presented with elevated levels of BPA and PFAS [213,214]. Similarly, an increase in serum PFAS was also believed to be the result of unregulated tubular reabsorption, thus extending the half-life of PFAS in the body [213]. As a result of EDC or EDC-metabolite accumulation, there is a potential to exacerbate any multi-organ, multi-system effects.

Since the kidneys serve a primary role in toxin and metabolite excretion, it is critically important to understand the effects of environmental exposures on kidney function. Classification of EDCs could help to identify at-risk patients in asymptomatic stages. While molecular mechanisms are still under investigation, there is sufficient evidence to support a correlation between environmental exposures and kidney dysfunction.

### 5.5. Respiratory Disease

Environmental chemicals have been known to cause adverse effects on human respiratory function since the Industrial Revolution accelerated the process of pollutant emission, and the hazards of tobacco products were acknowledged by the medical community. Indeed, air pollution is a major cause of lung cancer, asthma, allergies, COPD and other respiratory disorders. Common household products, including cleansers, soaps, and cosmetics, have recently come under scrutiny in relation to respiratory health. Over 50 EDCs and compounds associated with asthma were identified in a study of 200 commercial products [215]. Currently, a significant amount of attention is focused on polyvinyl chloride (PVC), one of the most widely produced synthetic plastic polymers. PVC exposure is ubiquitous, as the chemical is used in pipes, floors, doors, windows and many other household products. PVC contains phthalates which can be released into the environment. PVC flooring in the home was found to increase the risks of asthma and allergic rhinitis in a child cohort [216]. Likewise, a meta-analysis of several adult epidemiological studies revealed a significant association between occupational exposure to PVCs and the onset of respiratory symptoms [217].

### 5.6. Neurological Effects

The elevation in cognitive, psychiatric and behavioral disorders over the past few decades has raised public concern over the potential effects of EDCs on neurological function [218]. Epidemiological data do indicate that early childhood exposure to pesticides or EDCs found in cleaning products may result in a range of developmental defects and functional shortages in childhood, including neurocognitive deficits and abnormal or borderline social behavior [219,220,221,222]. One potential mechanism for neural effects of EDCs is through dysregulation of thyroid hormone signaling, which is required for proper development. Recently, it was reported that human amniotic fluid containing an EDC mixture was able to alter thyroid hormone action and brain development in a vertebrate model [223].

There also exists concern over potential effects of environmental chemicals on IQ and learning. Children living in residential areas with high levels of EDCs (including BPA, phthalates and others) possessed lower grade point averages [224]. Additionally, several studies have linked exposures to PCBs during fetal development to lower IQ [12].

Autism spectrum disorders (ASD) and attention deficit hyperactivity disorder (ADHD) have increased in the last few decades, prompting examination of the potential role of the environment as a causative factor. Epidemiological data, however, are conflicting. Three clinical reports positively correlate incidence of ASD with developmental exposure to BPA and phthalates, or contact with air pollution and pesticides [225,226,227], yet, the Health Outcomes and Measures of the Environment (HOME) study identified no relationship between EDC levels in 175 pregnant women and autistic traits in their children [184]. Further studies will be required to determine what, if any, impact EDC exposure may have on human neurological function, particularly the consequences when experienced in utero.

## 6. Conclusions

EDCs are ubiquitous and persistent in our environment. Detectable levels of numerous EDCs exist in human body fluids and tissues. Experimental data have established a strong causal link between EDC exposure and disorders of numerous organ systems and metabolic processes. Epidemiological studies have highlighted the clinical consequences of EDC exposure during various phases of development and adulthood. Since available human data are correlative, further studies will be required to establish a firm causal relationship between EDC exposure and human disease. However, there is sufficient experimental information on disease risks that can be extrapolated to predict human outcomes, and thus prompt us to limit EDCs exposure, especially during sensitive developmental periods (i.e., in utero, neonatal, and puberty). The ubiquitous nature of EDCs in our environment means that all humans are subject to these chemicals, and the NHANES studies described above have found detectable levels of EDCs in the vast majority of the United States population. There is currently less information available about EDCs in under-developed economies, highlighting the need to develop world-wide surveillance programs and unified regulations on use and dissemination.

While exposure to EDCs cannot be entirely avoided, it can be minimized, as noted below.

- Replacing plastics, particularly in food preparation, heating and storage with glass can greatly minimize consumption of phthalates, BPA, and related BPS and BPF. Diligence should be used in selection of BPA-free products, especially for infants, children, and pregnant women.
- There should be discrimination in the use of topical skin care products, particularly sunscreens, most of which contain the ultraviolet chemical filter oxybenzone (benzophenone-3; BP-3). BP-3 is an established EDC that shows remarkable systemic absorption [228], and which was recently detected in 97% of the population in a Center of Disease Control study [229]. Mineral-based sunscreens, containing zinc oxide or titanium dioxide as active ingredients, are now recommended over others.
- Well water should be tested annually. The use of filtered water will also minimize phthalate consumption.
- Pesticide ingestion can be reduced by consumption of organic produce or by soaking non-organic produce in a sodium bicarbonate solution.
- Minimize use of bleached paper products such as disposable diapers and paper towels, which contain dioxins
